# Surgical Cases

**Published:** 1866-08

**Authors:** Geo. K. Amerman

**Affiliations:** Attending Surgeon to Cook County Hospital, Chicago


*Surgical Cases.* By GEO. K. AMERMAN, Attending Surgeon to Cook County Hospital, Chicago.

CASE I. DOUBLE AMPUTATION FOR RAILROAD INJURY—PATIENT SITTING UP ON THE SEVENTEENTH DAY—DISCHARGED CURED ON THE FORTY-SECOND DAY.

On Friday, April 13th, 1866, Mr. H., a drover of this city, was accidentally run over by the cars. He was immediately carried to his hotel, and I saw him in less than an hour after the accident occurred. He was a large muscular man, 37 years old, of perfectly temperate habits, and had never been sick enough to require a physician in his life. The injury was entirely confined to his feet and legs. The left foot, ankle and lower third of the leg were crushed into one mass. The right ankle and lower third of the right leg were in the same condition. The right foot uninjured. There was scarcely any hemorrhage, and only a very moderate shock to the nervous system.

From the nature and extent of the injury, it was impossible to save either limb, and although double primary amputation gave very little hope of recovery, it being the patient's only chance, he readily assented to have the operation performed.

After a delay of about three hours, arranging preliminaries and waiting for reaction, the patient was fully etherized by Dr. Hutchinson, House Physician to Cook County Hospital, and with the assistance of Drs. Bingham and Marguerat, of this city, both legs were amputated by the double-flap method, about three inches below the knee-joint. Very little blood, not over four ounces, was lost during both operations. Simple dressings—sutures, a compress and roller—were applied, and the stumps so arranged on a pillow, that cold water could be showered over them without soiling the bed. Ordered morphia, sufficient to procure rest, and cold water to be applied when the parts became hot and painful.

April 14th. Patient has been quite comfortable since the operation. Slept some last night. Has very little pain. Pulse 108. Continue anodyne and cold water dressings.

April 18th. Not a single unfavorable symptom from the first. Pulse 106. Reapplied dressings for the first time. Both stumps looking well and partly united by adhesion. Discontinue cold water dressings, anodyne as usual.

May 1st. Seventeenth day. Found the patient sitting up, and says he feels as well as ever. Pulse 96. Appetite good, bowels regular, and sleeps well at night.

No treatment, except to dress the limbs every second day. Only a part of either wound healed by first intention. The remainder is granulating finely.

May 25th. Forty-second day after the operation. Patient entirely well and attending to business.

*Remarks.*—The favorable termination in the above case renders it one of very great interest. Such a result was wholly unexpected, and so far as my own experience and knowledge on this subject extend, it is the only successful case of double primary amputation for railroad injury on record. Several years ago, I performed the same operation under similar circumstances, but the patient only survived twenty-four hours.

This unexpected result depended mainly upon the condition of the patient at the time of the operation and his previous habits. He was 37 years old, and had never been sick a day in his life—was perfectly temperate, never drank a glass of liquor, and very rarely a cup of coffee or tea, and all his habits of life were regular and favorable to perfect health. All this exerted a powerful, and no doubt the chief, influence in his recovery, but it also depended in some degree, we believe, upon the operation and subsequent treatment. In our own mind, three circumstances attending the operation contributed to its success. First—waiting until reaction was fully established. This we conceive to be of the very first importance, and too often overlooked in our anxiety to relieve our patient and get rid of mangled and unsightly parts. Second—the small amount of blood lost during the operation. This, too, is all important, and should be carefully attended to in every case. Take time to apply the tourniquet accurately and effectually, first elevating the limb that the venous blood may empty into the large abdominal trunks, and every ounce of blood saved will help your patient in his recovery. And lastly—the removal of all muscular and tendinous tissue from the flaps, forming them entirely of skin and subcutaneous cellular tissue. This procedure greatly diminishes the subsequent suppuration and perhaps the twitchings and unpleasant motions of the stump. A matter like this seems of very trivial importance, and yet it is by attending to the "little things" that we shall achieve our greatest success.

In the subsequent treatment, two circumstances contributed to the successful result. First—an abundance of fresh air. The room was kept open, and a current of air passed through it day and night. And second—opiates sufficient to relieve pain and procure rest. Whenever the patient began to complain of pain, and became restless and uneasy, opium was given at once, and enough to make him quiet. It seemed to have a most beneficial effect and contributed greatly to his recovery.

CASE II. EXCISIONS OF THE SHAFT OF THE HUMERUS—DELIRIUM TREMENS—RECOVERY WITH A USEFUL ARM.

On Sunday, August 13, 1865, Mr. H., a large healthy German, was shot in a row. The ball entered the left arm at about its middle, anteriorly, and passing through the humerus, made its exit directly opposite, posteriorly. The soft parts were immensely swollen when I saw him the day after the injury, and presented a dark livid appearance. The humerus was extensively fractured and comminuted. The vessels and nerves apparently uninjured.

The age of the patient, his general good health, and the apparent safety of the vessels and nerves, decided the operation of excision in preference to amputation, and with the assistance of Drs. Bogue and Smith, of the Hospital staff, the patient was anæsthetised, and about four inches of the middle of the shaft of the humerus, together with all loose fragments, were removed. The wound was closed with interrupted sutures, the arm laid on a pillow, temporarily, and cold water dressings constantly applied.

August 15. Patient restless and unable to sleep—thirst—no appetite. Pulse 120. Arm looking well, but immensely swollen. Began to fear delirium tremens, as the patient had been on a spree for the last three weeks, and at once ordered opium in large doses.

10 P. M. Patient perfectly uncontrolable, removing every particle of dressing from the arm, getting up and swinging it over his head, declaring there is nothing the matter with him. The symptoms were all decidedly unfavorable. Pulse feeble and too rapid to count. Countenance haggard and sallow. Ordered opium, grs. iij. every hour, given in lager beer to get it down.

August 16, 8 A. M. Patient sleeping quietly and sweating profusely. Pulse 110. Placed the arm in an angular splint and reapplied cold water dressings. Ordered nourishment and quinine.

August 22. Doing well. Appetite good. Pulse 90. Sleeps well, and every symptom favorable. Wound granulating and closing nicely.

September 2. A small piece of bone came away with the discharges this morning.

October 16. Wound entirely healed, and considerable formation of new bone. From this time onward, the case progressed without an unfavorable symptom. The arm became stronger, daily, and about the first of November, three months after the operation, all dressings were removed, and the patient advised to begin its moderate use.

June 1st, 1866. Patient able to attend to business, and has very good use of the arm. The result was not quite as favorable as anticipated, on account of a second row he indulged in —refracturing the bone soon after the union became firm.

*Remarks.*—The interesting feature connected with the above case, is the favorable result, notwithstanding all the untoward circumstances attending the after treatment. The operation of excision in the continuity of the long bones of the extremities, is still of rather doubtful expediency; and every case, whether successful or not, will be important to aid in deciding between excision and amputation.

In the Reports of the Surgeon General of the U. S. A., the total number of cases of excision of the shaft of the humerus was 261, of which 42 died, 133 recovered, 7 were subsequently amputated, and in 79 the result was undetermined. The Surgeon General remarks, that "after excisions of portions of the shaft of the humerus for gunshot fractures, a number of patients have certainly obtained very useful limbs. But the mortality after the operation is three per cent. greater than after amputations of the arm. The 52 preparations at the Army Medical Museum, illustrating this resection, indicate the frequency with which it is followed by secondary amputation or a fatal result."

